# Could giardiasis be a risk factor for low zinc status in schoolchildren from northwestern Mexico? A cross-sectional study with longitudinal follow-up

**DOI:** 10.1186/1471-2458-10-85

**Published:** 2010-02-20

**Authors:** Luis Quihui, Gloria G Morales, Rosa O Méndez, Johanna G Leyva, Julián Esparza, Mauro E Valencia

**Affiliations:** 1 Department of Public Nutrition and Health, Centro de Investigación en Alimentación y Desarrollo A. C. Hermosillo, Sonora, México

## Abstract

**Background:**

Both giardiasis and zinc deficiency are serious health problems worldwide. In Mexico, the prevalence of *G. intestinalis *was estimated at 32% in 1994. It remains a health problem in northwestern Mexico. Recent surveys (1987, 1995, and 1999) reported zinc deficiency in the Mexican population. The association of giardiasis and malabsorption of micronutrients has been well documented, although the association with zinc remains controversial. This study investigated the association between giardiasis and zinc deficiency in schoolchildren from northwestern Mexico.

**Methods:**

We combined a cross-sectional design with a longitudinal follow-up six months after parasite treatment. The baseline sample consisted of 114 schoolchildren (mean age 8.8 yr) from seven suburban public schools, grouped as *Giardia*-free (*n *= 65, 57%) and *Giardia*-infected (*n *= 49, 43%). Three stool analyses per child were done using Faust's method. Children with giardiasis received secnidazole. Serum zinc was determined by atomic absorption spectrophotometry. Height and weight were measured. Socioeconomic information was obtained in an oral questionnaire, and daily zinc intake was assessed using 24 hour-recalls. Pearson's correlation and ANCOVA and paired t-test analyses were used to determine the association between giardiasis and zinc status.

**Results:**

Longitudinal analysis demonstrated a significant increase of the mean serum zinc levels in the *Giardia*-infected group six months after treatment (13.78 vs. 19.24 μmol/L μmol/L; p = 0.001), although no difference was found between the *Giardia*-free and the *Giardia*-infected groups (p = 0.86) in the baseline analysis. Z scores for W/A and H/A were lower in the *Giardia*-infected than in the *Giardia*-free group (p < 0.05). No difference was observed in the socioeconomic characteristics and mean daily intakes of zinc between the groups (p > 0.05).

**Conclusions:**

Giardiasis may be a risk factor for zinc deficiency in schoolchildren from northwestern Mexico.

## Background

Giardiasis, a worldwide public health problem, is caused by *Giardia intestinalis *(*G. intestinalis*). In 2001 [1] and 2004 [2], a billion cases and a global prevalence of 30% were estimated respectively. Current worldwide prevalence among children under 10 yr of age range from 15% to 20% [2]. Low levels of education, poor hygiene, poor drinking water, overcrowded conditions, and poor sanitation increase the prevalence of giardiasis [3-5].

Zinc deficiency is another increasing public health problem. In 2004, its global prevalence was estimated at 31%, ranging from 4% to 73% across developing countries [6]. Zinc deficiency has been found to be caused by poor intake and malabsorption, and has been associated with growth retardation, neuro-sensory changes, impaired cognitive function, abnormal immune functions, and death [7-9].

The association between zinc deficiency and infection has scarcely been investigated [10], although the association of *G. intestinalis *with malnutrition [11] and malabsorption of micronutrients such as vitamin A [12,13] is well recognized. In 1993, [14] giardiasis was reported as a first-time risk factor for zinc malabsorption in children. Other authors reported this risk [15-17]. However, the link remains controversial [18]. In Mexico, the prevalence of *G. intestinalis *was estimated at 32%, in 1994 [19]. Currently *G. intestinalis *is the most important protozoan parasite associated with intestinal infection in northwestern Mexico [20-22]. Three studies in Mexico have shown evidence of zinc deficiency in women and children [23-25]. One study showed low dietary zinc consumption in 19% to 24% of schoolchildren [25].

We hypothesized that giardiasis may be a risk factor for zinc deficiency in Mexican schoolchildren under poor socioeconomic and environmental conditions. The aim of this study was to investigate the association between giardiasis and serum zinc levels in schoolchildren from northwestern Mexico. Specific objectives included measurements of a) serum zinc levels in *Giardia*-free and *Giardia*-infected children, and b) relating nutritional status to growth, weight, diet, and environmental and socioeconomic factors.

## Methods

### Study design

This study was cross-sectional with a longitudinal follow-up. Data collection, collection and processing of blood and stool samples and anthropometry measurements were performed at baseline and repeated six months after treatment.

### Study population

The study sample consisted of primary schoolchildren from grades one to six at seven suburban public schools in the cities of Hermosillo and Guaymas, in northwestern Mexico. The study sites selection was based on the high frequency of giardiasis associated with morbidity in the general population of these cities [21,22], and the low socioeconomic level of the populations at the study sites [26-28]. We explained the purpose of the study at official meetings with personnel from health services, city councils, and the primary schools, and at meetings with parents and schoolchildren from the study sites.

A total of 1,672 schoolchildren between the ages of six yr and ten yr were officially enrolled in the visited schools [27] and they were invited to join the study, while distribution of plastic containers with requests for fecal samples were undertaken (three from each subject were to be collected in the school during a five day period). Only 293 (18%) schoolchildren gave samples, but 114 children met the study criteria (voluntary participation supported by their parents, no fever, respiratory infections or diarrhea, and no zinc supplementation and/or antiparasitic treatment). Eighteen (6%) children were excluded because they gave fewer than three fecal samples; 59 (20%) were excluded because they were infected not only with *G. intestinalis *but also with other pathogenic parasites (*H. nana *and *E. histolytica/dispar*); 41 (14%) were excluded because they were currently supplemented; 25 (9%) had infections [29]; 24 (8%) were unwilling to participate, and 12 (4%) did not complete the study. When required, the excluded children (57%) were referred for medical attention and proper treatment. Written consent from parents or guardians was obtained for the included children to participate. Approval to conduct the study was granted by the ethical committee of the Centro de Investigación en Alimentación y Desarrollo A.C. (CIAD AC).

### Stool analysis

Stool samples were examined using the Faust technique [30]. An experienced parasitologist performed the analysis and microscopic observations.

### Anti-parasitic treatment

Secnidazole was orally administered, in a 450 mg dose-per-day for two successive days, to *Giardia*-infected schoolchildren. Treatment was repeated when necessary to ensure elimination of the *Giardia *infection [31]. A qualified physician prescribed the treatment.

### Anthropometry

Height and weight were measured in 98 schoolchildren at baseline and six-month follow up. These measurements could be only undertaken at baseline in the rest of the children (n = 16) because they moved out from their schools during the study course. Height was measured using a stadiometer (Seca) marked at 0.1 cm intervals, and weight was measured to the nearest 50 g using a digital electronic scale (AND FV-150 KA1, A&D Co. LTD, Japan). The children were measured without shoes and wearing light clothing. For weight measurement, each child stood unassisted in the centre of the platform scale and was asked to look straight ahead and relaxed. Standing height was measured when the child stood straight with the head in the Frankfurt plane, shoulders in relaxed position, and arms hanging loosely [32]. Ages of the children were verified from birth certificates. Undernutrition was defined as below -2 standard deviation units (-2 Z Scores) from the median reference values defined by the World Health Organization [33], using the nutritional indices of height for age (H/A; stunting), weight for age (W/A), and weight for height (W/H; wasting).

### Socioeconomic data

Collection of the socioeconomic characteristics of the children's families was undertaken with a structured and locally pilot-tested questionnaire [34]. The interviews were administered face-to-face with mothers in the children's schools. A local leader trained the interviewers to lessen bias. Socioeconomic status was assessed from the employment status and education of the parents, assigning (0) for unemployed or (1) for employed, and (0) for incomplete or (1) for complete secondary school. Household conditions were assessed by the type of material used for walls, roofs, and floors, categorized on the basis of local costs. Sanitation facilities were assessed as defecation in an open area (0), or the use of a pit/latrine (1); drinking water was assessed as tap water (0), or treated water (chlorine/boiled) (1). Crowding was estimated using the number of people per room, and categorized as less (0) or more (1) than five people per room. Family income was estimated as number of minimum daily-wages, dividing the daily family income by the current local minimum daily wage [35].

### Dietary zinc

A trained interviewer assessed each child's daily intake of zinc using the 24-hour recall method. The children were interviewed twice in the presence of the mother. The first interview occurred at the time of the blood sample collections. It was repeated six months later. Dietary intakes of 4 mg of zinc per day are recommended for children of ages 6-8 yr; and 7 mg for ages 9-12 yr [36]. Dietary intakes were categorized as (1) for intakes equal/above and (0) for intakes below the recommendation.

### Blood sampling

A 10 mL sample of venous blood was taken from the cubital vein of each child using winged set of needles (23 × 19 mm) and Vacutainer™ glass tubes (13 × 100 mm) with SST Gel & Clot activator to separate the serum fraction. Within 2 hr, blood samples were transported to the laboratory and centrifuged at 1100 g for 10 min. Serum was separated, labeled, and stored at -70°C, awaiting zinc determination.

### Determination of serum zinc

Serum zinc was determined by atomic absorption spectrophotometry, using the procedure recommended by the AOAC. Measurements and analysis were developed and carried out by a qualified technician [37]. A sample of 0.40 mL of serum was diluted with 2.0 mL of 0.03% brij solution 35. A certified sample of non-fat Milk 1549 (NIST SRM) was used as an external control, with a mean of 46.1 ± 2.2 μZn/mL in the interval of confidence of 95%, before the analysis of each blood-sample-set of 50. Measurements were carried out at 213.9 nm using a hollow-cathode zinc lamp with a coefficient of variation (CV) of 2.6%, and a recovery of 97%. The cut-off point for zinc deficiency was set at < 10.7 μmol/L [38].

### Statistical analysis

Data were analyzed using NCSS 2000 software (NCSS Statistical Software, Kaysville, UT). The distribution of each variable was tested for normality before the analysis using the Kolgomorov-Smirnov goodness of fit test. When necessary, data were normalized using the logarithmic transformation. Descriptive statistics were expressed as mean ± standard deviation or geometric mean (± SE) for skewed continuous variables, and percentage for categorical variables. Z scores were evaluated by anthropometrical software, version 1.01, using data from the National Center for Health and Statistics as recommended by the World Health Organization [39]. Cross-sectional data were assessed by independent sample *t*-tests. Pearson's correlation test was used to examine the association between the independent and dependent variables. Analysis of covariance (ANCOVA) was used to compare the means of Z scores between the groups, controlling for the variables that had p ≤ 0.15 in the Pearson's correlation analysis. The chi-square test was used to test the significance of differences in frequency distributions between the *Giardia*-free and the *Giardia*-infected groups. In the longitudinal analysis, paired t-test was used to compare the means of the serum zinc levels of the *Giardia*-infected group before and after treatment. The same analysis was applied to compare the serum zinc levels of the *Giardia*-free group. All analyses were considered significant at p ≤ 0.05. In addition, multiple linear regressions was used to test whether difference between the serum zinc levels at baseline and six months after treatment between the *Giardia*-infected and *Giardia*-free groups adjusted by the initial zinc values. An interaction term between the initial zinc values and *Giardia *infection (group variable) was also tested using a p-value of 0.1

## Results

At baseline, the average age of the schoolchildren (n = 114) was 8.8 (1.0) yr. Fifty-four (54%) were girls. The proportions below -2 SD in W/A and H/A (stunting) were 7%, and 16.7%, respectively. No cases of wasting (W/H) were detected. Means for W/A, H/A and W/H were -0.05 (1.3), -0.9 (1.1) and 1.0 (1.6), respectively. No significant differences were found between the boys and the girls (p > 0.05) (data no shown).

The mean ages of the *Giardia*-free and the *Giardia*-infected groups were 8.8 (0.9) yr and 8.7 (1.1) yr, respectively. W/A and H/A were significantly higher in the *Giardia*-free than in the *Giardia*-infected group (p < 0.05). No difference was found for W/H between the groups (p = 0.30) (Table 1). There was no difference in the mean daily intakes of zinc between the *Giardia*-free and the *Giardia*-infected groups (p = 0.12) (Table 1).

**Table 1:** The physical characteristics and daily zinc intakes of the *Giardia*-free and *Giardia*-infected groups of schoolchildren at baseline.

Variables	*Giardia*-free	*Giardia*-infected	*p value
	(n = 65)	(n = 49)	
Age (yr)‡	8.8 (0.9)	8.7 (1.1)	0.70
Weight (kg)†§	30.5 (1.2)	27.1 ± (0.9)	0.02
Height (cm)†	128.7 ± 6.9	125.5 ± 7.4	0.03
W/A (z-score)†	-0.2 ± 1.3	-0.4 ± 1.3	0.04
H/A (z-score)†	-0.70 ± 1.0	-1.2 ± 1.1	0.02
W/H (z-score)^∞^	1.2 ± 1.7 47	0.8 ± 1.5 38	0.30
Daily zinc intake (mg/day)‡	5.9 ± 1.9	8.3 ± 11.7	0.12

Mean ± SD; ‡ n = 114: † n = 94; ^∞ ^n = 85; § Geometric mean (Standard Error); * Independent t-test

In the *Giardia*-infected group no significant differences were found between values before and values six months after treatment for the mean daily zinc intakes (8.30 mg vs. 6.01 mg respectively) (p = 0.19).

About 91% of the fathers (n = 104) and 28% of the mothers (n = 32) had formal jobs at the time of the interviews. The fathers represented the family economic support. Secondary education was completed by more than 50% of the children's parents. Chi-square analysis showed no significant difference between the *Giardia*-free and the *Giardia*-infected groups in the employment status (p = 0.85 and p = 0.96 between mothers and between fathers respectively) and level of education of the parents (p = 0.86 and p = 0.97 between mothers and between fathers respectively), the quality of walls and roof (p = 0.51), quality of floor (p = 0.94), the types of drinking water (p = 0.48), overcrowding (p = 0.77) or the family income (p = 0.22) in the cross-sectional analysis (data no shown).

**Figure 1:**
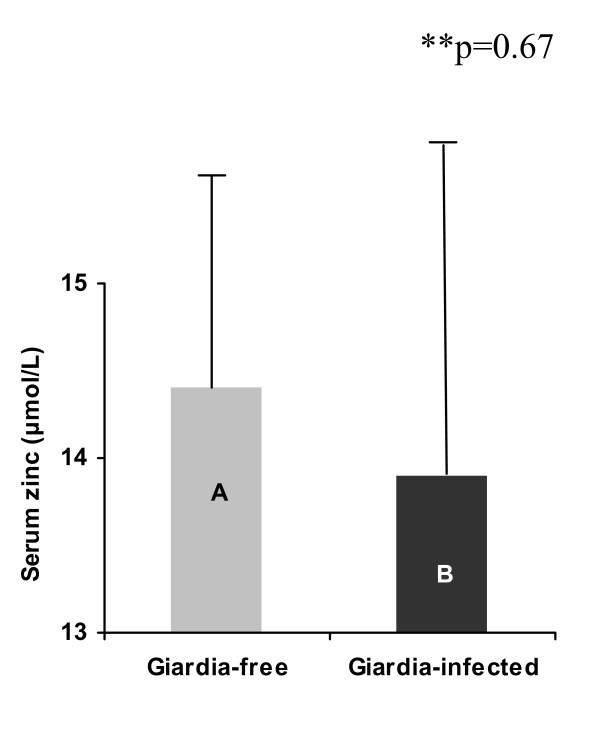
**Comparison of the serum zinc levels between the *Giardia*-free (n = 65) and the *Giardia*-infected (n = 49) groups (cross-sectional analysis)**. A = 14.4 (1.4), *B = 13.8 (1.9), *Error Bars = (S.E.). * Non-adjusted geometric means. ** Adjusted ANCOVA (region, sex, age and intake of zinc).

**Figure 2:**
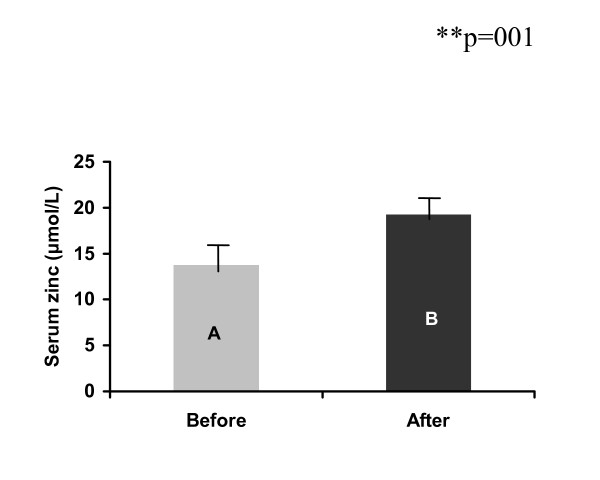
**Comparison of the serum zinc levels before (n = 49) and six months after treatment (n = 49) in the *Giardia*-infected schoolchildren (longitudinal analysis)**. A = 13.8 (1.9), *B = 19.2 (0.9), *Error Bars = (S.E.). * Non-adjusted geometric means. ** Adjusted ANCOVA (region, sex, age and intake of zinc).

At baseline, independent t-test showed no difference (p = 0.67) in the geometric means (± SE) of the serum zinc levels 14.46 (0.79) and 13.78 (1.33) μmol/L between the *Giardia-free *(n = 65), and *Giardia*-infected children (n = 49) respectively. When controlling for region, sex, age and daily zinc intake using ANCOVA analyses the same result was found (p = 0.98) (Fig. 1). Four children from the *Giardia*-free and seven from the *Giardia*-infected group had serum-zinc levels below the cut-off value, 10.7 μmol/L [37]. However, six months after treatment, a significant increase in the geometric mean of serum zinc was found in the *Giardia*-infected group (13.78 vs. 19.24 μmol/L) (p = 0.001 from paired t-test analyses) (Fig. 2). After treatment, no children showed a serum zinc concentration below the cut-off value. Conversely, although it was not significant, the serum zinc levels showed a tendency of increasing in the *Giardia*-free group after six months (14.46 μmol/L vs. 16.98 μmol/L respectively; p = 0.08). In addition, comparison of the difference between the serum zinc levels at baseline and six months after treatment between the *Giardia*-infected and *Giardia*-free groups adjusted by the initial zinc values using multiple linear regression, showed a significant larger increased serum zinc values in the *Giardia*-infected group than in the *Giardia*-free group (β = 2.54, CI = 1.02- 4.15, p = 0.001). Since the reference group in the regression model was the *Giardia*-free, the positive β-coefficient value means that the increase in serum zinc level was 2.54 μmol/L larger in the *Giardia*-infected than that in the *Giardia*-free group adjusted by the initial zinc values. No interaction was observed between the initial zinc values and the *Giardia *infection or group variable (β = -0.15, CI = -0.35 - .06, p = 0.16).

## Discussion

Thirty five percent of the *Giardia*-free and 29% the *Giardia*-infected children showed daily zinc intakes below the recommended values [36]. In 1999, the National Survey of Nutrition estimated that about 60% of the general population of Mexico and 57% the northern region had daily zinc intakes below the recommendation [25]. The present study suggests that schoolchildren in northwestern Mexico have fewer incidences of zinc-deprived diets than the general national population. Low levels of parental education, poor income and household conditions, high overcrowding, poor sanitary conditions, and low quality of drinking water are associated with a high prevalence of intestinal parasitic infections [4,40]. Our study population was attending public schools located in underprivileged areas, and no differences were found in the socioeconomic variables between the *Giardia*-free and *Giardia*-infected groups.

A Mexican study revealed a higher prevalence of parasitic infections in 65 out of 372 (22.5%) children with lower nutritional status, compared to 32 out of 285 (11%) in better-nourished schoolchildren [41]. In 2005, another study in Turkey [42] showed higher Z scores for H/A and W/A in a *Giardia*-free group than in the corresponding group of *Giardia*-infected schoolchildren. Intestinal parasites are associated with childhood malnutrition, where malnutrition may increase the susceptibility to parasites, or parasites may deteriorate the nutritional status of the host [2,43]. In our study, the children free of *Giardia *and/or other pathogenic parasites showed significantly higher Z scores for W/A and H/A than the *Giardia*-infected children.

In the present study, no cases of wasting were observed, in agreement with the National Survey of Nutrition in 1998 [44]. Probably, in regions with both chronic nutrient deficiency and infections, children adapt their stature to their lower weight. In this way, children may appear to have a normal W/H, but in reality, they are children of low stature. This condition is referred as "homeorrhesis" [45].

One published Mexican study [24] has shown that 20% of 219 schoolchildren from rural southern Mexico had serum zinc levels less than 1.6 μmol/L. This suggested that our schoolchildren are less nutritionally zinc-deprived than those from the southern region, who probably consume less dietary zinc. In 1999, 66% of the southern general population did not meet the daily zinc recommendation, and 34% of the children under five yr old showed zinc deficiency [25].

In the present study, giardiasis was the difference in the observed results as evidenced by the parasitic treatment. This finding is supported by both the non-significant increase in the mean serum zinc levels from baseline to six months after in the *Giardia*-free group and the significant larger increased serum zinc value observed in the *Giardia*-infected group than in the *Giardia*-free group. Although the association between malabsorption and giardiasis is well documented, little is known about the giardiasis-zinc interaction. Recently, two studies from Turkey [15,16] showed that *Giardia*-infected groups of children (45 and 34 respectively), 2 yr to 14 yr old, had lower mean serum zinc levels than their matched *Giardia*-free group (10.3 μmol/L vs. 220.2 μmol/L, and 16.7 μmol/L vs. 20.8 μmol/L, respectively). Another study from Turkey [17] found a significant increase in the mean serum zinc levels after treatment (8.7 μmol/L vs. 14.8 μmol/L) in 20 *Giardia*-infected children of ages 3 mo to 14 yr. In contrast, a Spanish study [18] found no change in the mean serum zinc levels before (14.1 μmol/L) and three months after treatment (14.1 μmol/L) in 25 *Giardia*-infected children of ages 6 yr to 9 yr. It must be remarked that the Turkish and Spanish children were from low and medium socioeconomic levels respectively, and the baseline means for serum zinc in the Turkish children were lower than in the Spanish children. The children in our study were from low socioeconomic level, and showed a mean serum zinc level at baseline similar to that of the Spanish children. In addition, the mean serum zinc levels were always above 10.7 μmol/L. On the other hand, it is possible that a no detected increase in zinc intake due to a raised awareness of the issue in mothers during the study course could have been related to the increased serum zinc levels in these children. However, recalls revealed no zinc supplementation or increased food intake in this study. In addition, the main foods recognized as major zinc contributors to the children's diets were milk, corn tortilla, ground beef, eggs, sausage, flour tortilla, beans, cheese and chicken, and they were invariably present in the recalls collected at baseline and follow-up.

How zinc metabolism is compromised by *G. intestinalis *is not well known, but it is hypothesized that the increased intestinal absorption of zinc associated with anti-*Giardia *treatment may be explained by the restoration of intestinal mucosa that had been impaired by giardiasis [46]. However, our cross-sectional data analysis showed no significant differences between the *Giardia*-free and the *Giardia*-infected groups in this study. Although some limitations in the cross-sectional analysis may explain these findings: a) The distinction between the *Giardia*-free and *Giardia*-infected groups may hide the real association between giardiasis and low serum zinc, because all the children could have been infected with *Giardia *sometime prior to this study. b) If *Giardia*-free children showed no infection because of recent treatment, their serum zinc levels might yet not have been restored to a point significantly different from that in the *Giardia*-infected children. c) The duration of the *Giardia *infection may not have been long enough to significantly decrease the serum zinc levels in the *Giardia*-infected children. d) The sample size may not have been large enough to find a significant difference between the groups. Despite this, the validity of this study was supported by the findings of the longitudinal data analysis in the *Giardia*-infected and *Giardia*-free groups.

## Conclusion

Causes of zinc deficiency may be multifactorial. However, results from this study show that giardiasis may be a risk factor for zinc deficiency in the schoolchildren from northwestern Mexico. Other studies are required to elucidate the pathological mechanism implicated in the zinc-giardiasis interaction. More information about zinc levels in the Mexican population is required to redesign current national strategies for parasitic control, micronutrient supplementation and food fortification, in order to improve the quality of life of vulnerable populations.

## Competing interests

The authors declare that they have no competing interests.

## Authors' contributions

LQ was the project leader and participated in the study design, sample and data collection, parasitological analysis, and writing of the manuscript. GGM performed sampling, data collection and preparation of the database and manuscript. ROM contributed with the study design, data interpretation and writing of the manuscript. JGL participated in the study design, sample and data collection, parasitological and biochemical analysis, and results discussing. JER carried out the statistical analysis, data interpretation and writing of the manuscript. MEV participated in the study design and revised manuscript. All authors read and approved the final manuscript.

## Pre-publication history

The pre-publication history for this paper can be accessed here:

http://www.biomedcentral.com/1471-2458/10/85/prepub
